# Configuring ADAS Platforms for Automotive Applications Using Metaheuristics

**DOI:** 10.3389/frobt.2021.762227

**Published:** 2022-01-04

**Authors:** Shane D. McLean, Emil Alexander Juul Hansen, Paul Pop, Silviu S. Craciunas

**Affiliations:** ^1^ Technical University of Denmark Kongens Lyngby, Kongens Lyngby, Denmark; ^2^ TTTech Computertechnik AG, Vienna, Austria

**Keywords:** automotive applications, task scheduling, task preemption, time-sensitive networking, TSN, IEEE 802.1Qbv

## Abstract

Modern Advanced Driver-Assistance Systems (ADAS) combine critical real-time and non-critical best-effort tasks and messages onto an integrated multi-core multi-SoC hardware platform. The real-time safety-critical software tasks have complex interdependencies in the form of end-to-end latency chains featuring, e.g., sensing, processing/sensor fusion, and actuating. The underlying real-time operating systems running on top of the multi-core platform use static cyclic scheduling for the software tasks, while the communication backbone is either realized through PCIe or Time-Sensitive Networking (TSN). In this paper, we address the problem of configuring ADAS platforms for automotive applications, which means deciding the mapping of tasks to processing cores and the scheduling of tasks and messages. Time-critical messages are transmitted in a scheduled manner via the timed-gate mechanism described in IEEE 802.1Qbv according to the pre-computed Gate Control List (GCL) schedule. We study the computation of the assignment of tasks to the available platform CPUs/cores, the static schedule tables for the real-time tasks, as well as the GCLs, such that task and message deadlines, as well as end-to-end task chain latencies, are satisfied. This is an intractable combinatorial optimization problem. As the ADAS platforms and applications become increasingly complex, such problems cannot be optimally solved and require problem-specific heuristics or metaheuristics to determine good quality feasible solutions in a reasonable time. We propose two metaheuristic solutions, a Genetic Algorithm (GA) and one based on Simulated Annealing (SA), both creating static schedule tables for tasks by simulating Earliest Deadline First (EDF) dispatching with different task deadlines and offsets. Furthermore, we use a List Scheduling-based heuristic to create the GCLs in platforms featuring a TSN backbone. We evaluate the proposed solution with real-world and synthetic test cases scaled to fit the future requirements of ADAS systems. The results show that our heuristic strategy can find correct solutions that meet the complex timing and dependency constraints at a higher rate than the related work approaches, i.e., the jitter constraints are satisfied in over 6 times more cases, and the task chain constraints are satisfied in 41% more cases on average. Our method scales well with the growing trend of ADAS platforms.

## 1 Introduction

Advanced Driver Assistance Systems (ADAS), present in more and more modern consumer vehicles, perform complex functions that range from driver assistance, e.g., automated or assisted parking, lane changing, etc., to fully autonomous driving. In modern ADAS systems, there is a drive towards moving functions from hardware to software and the architecture from distributed to centralized, allowing modularization within an integrated hardware platform that can be cooperatively used and centrally managed (Niedrist, 2018). This drive has multiple advantages, like reusability and portability, but presents several challenges, especially in terms of real-time, testing, and safety (Gietelink et al., 2006). The fusion of multiple software functions of different criticality levels onto the same hardware platform has to be done in a composable manner with guaranteed temporal and spatial isolation without sacrificing real-time capabilities. This mixed-criticality paradigm applied to the automotive domain requires new concepts in terms of safety-critical temporal and spatial isolation, new scheduling results and configurations tools, as well as analysis methods for SIL certification (c.f. (Hammond et al., 2015; Niedrist, 2018)).

Generally, integrated ADAS platforms are composed of heterogeneous multi-core CPUs and Systems-on-chip (SoCs) of different performance and safety levels that are interlinked by a (real-time) communication network (Sommer et al., 2013; Becker et al., 2016b). In such integrated platforms, the ADAS functions have complex timing requirements and feature a complex interdependence between sensors, control software, and actuators. For example, one function for driver assistance collects sensor data from both cameras and distance sensors (ultrasonic, LIDAR) into a sensor fusion layer which transmits the data via the time-aware communication backbone to control algorithms that activate, e.g., the emergency brake system. This succession of function execution and message transmission creates a temporal dependency chain, which has to comply with a set of timing requirements in terms of latency. In order to guarantee both the interdependence and real-time behavior of tasks and their messages, the safety-critical ADAS functions and their communication frames have to be scheduled appropriately. Moreover, other less critical systems, like infotainment, are also integrated into the same platform and must not interfere with the real-time behavior of critical functions.

### 1.1 Related Work

The scheduling of task sets with dependencies has been a well-studied topic within the real-time community. Task schedules with inter-task dependencies are computed in (Chetto et al., 1990) by modifying the offsets and deadlines of the individual tasks and then using EDF to schedule the new task set (Buttazzo, 2011). In (Choi and Agrawala, 2000) the notion of absolute and relative timing constraints (i.e., events are temporally dependent on each other) for source and sink task requirements are introduced. Furthermore, the authors present a scheduling approach for uniprocessor systems with complex timing constraints such as jitter requirements. In (Fohler, 1994) the authors compute static schedules for tasks that communicate through bounded delay protocols like TDMA or TTP using dependency graphs. The work in (Tindell and Clark, 1994) presents an analysis of the schedulability of tasks that communicate using the TDMA protocol. In (Abdelzaher and Shin, 1999) an optimal task schedule for communicating tasks is generated using a branch-and-bound approach. A similar approach is introduced in (Peng et al., 1997) with modified optimization criteria. (Craciunas et al., 2014) presents a similar heuristic scheduling approach to ours which uses EDF simulation to create static schedules for tasks with communication and precedence dependencies but in contrast to our work, the results only apply for dependencies between tasks with equal periods. The temporal dependencies between tasks presented in the prior work described above are not as complex as the ones arising from the ADAS task chains where not only task periods can be different, but the correctness of the chain dependency is related to individual task jobs. Multi-rate tasks and complex precedence constraints have been analyzed in (Forget et al., 2011, 2017; Mubeen and Nolte, 2015). Additionally, in (Isović and Fohler, 2000), a two-step approach for distributed systems is introduced, which is based on an offline computation and an online EDF mechanism for scheduling tasks with complex constraints like jitter and job-level precedence requirements.

In the context of ADAS, the complex task chain requirements have been addressed in terms of computing the worst-case end-to-end latency of multi-rate chains, c.f. (Becker et al., 2017), depending on the available system information, e.g., scheduling algorithm or task offsets. Our approach is different in that it generates schedules that already adhere to the task chain requirements, which does not necessitate a further analysis since the schedule construction guarantees the real-time requirements. In (Rajeev et al., 2010), the authors present a model-checking-based method to compute worst-case response times and end-to-end latencies of tasks that have chain dependency and communication constraints. In (Becker et al., 2016a), the authors introduce a task chain latency analysis that does not require information about the concrete scheduling algorithm. (Verucchi et al., 2020) use an existing list-scheduling algorithm but apply it on a directed acyclic graph (DAG), which is constructed from multi-rate task sets such that complex precedence and timing constraints are captured and satisfied.

In (Lukasiewycz et al., 2012) a modular framework for ILP-based scheduling of time-triggered distributed automotive systems is presented, where both bus access and operating system schedules are created. The end-to-end latency of chains only applies single-rate dependency chains, and the method suffers from an exponential increase in runtime with increasing the number of tasks and messages. An extension that adds an incremental step in order to reduce the runtime complexity of the schedule generation has been proposed in (Sagstetter et al., 2014), where the focus is on integrating locally optimized schedules into a globally non-optimal solution. In terms of the communication backbone, the scheduling problem for TSN networks has been addressed in, e.g., (Craciunas et al., 2016; Serna Oliver et al., 2018) for fully deterministic communication needs, including latency and jitter requirements without taking into account the schedule of the communicating tasks. Furthermore, the combined task and message scheduling problem has also been thoroughly studied for other types of networks, e.g., TTEthernet (Craciunas and Serna Oliver, 2016), shared registers (Becker et al., 2016a), or the Universal Communication Model (Pop et al., 2003).

### 1.2 Contributions

In our earlier work (McLean et al., 2020) we considered that the communication backbone is done via Peripheral Component Interconnect Express (PCIe), and we have used a periodic real-time task model in which the worst-case execution time (WCET) of a task changes based on the core speed and the communication is modeled as overhead at the end of task instance execution. This paper extends our work to consider the IEEE 802.1 Time-Sensitive Networking (TSN) deterministic Ethernet standard for the communication. TSN is becoming a de-facto standard in several areas, e.g., industrial, automotive, avionics, space, with a broad industry adoption and several vendors developing TSN switches. This paper presents a heuristic-based scheduling algorithm for ADAS platforms that considers the different dimensions of timing and dependency requirements and is designed with scalability in mind. The optimization approaches are based on metaheuristics (Simulated Annealing and Genetic Algorithm), which take into account not only the timing constraints but also design goals, such as function allocation on computing units. We consider both PCIe and TSN for the communication. Future work may also include the LET model (Biondi and Di Natale, 2018) which is becoming increasingly popular in the automotive domain since it can provide deterministic communication behavior.

To the best of our knowledge, this is the first work to propose a heuristic-based solution to the combined scheduling problem in ADAS platforms that requires a solution for the task-to-core assignment, static task schedule generation, and the scheduling of TSN messages sent by the tasks, which respects both task and complex task chain timing constraints.

We start by introducing the platform and application models in Section 2 followed by a description of the scheduling problem in Section 3. We introduce the algorithm in Section 4 followed by an experimental evaluation in Section 5 and conclude the paper in Section 6.

## 2 Platform and Application Models

### 2.1 System Model

The modern integrated ADAS hardware platform features a multi-core multi-SoC embedded ECU with a variety of CPUs and Graphics Processing Units (GPUs) running at different speeds, which are interconnected through either a deterministic Ethernet backbone, such as TSN (IEEE, 2016b) or TTEthernet (Steiner et al., 2011), or through PCIe. RazorMotion (TTTech Computertechnik AG, 2018), for example, features a Renesas RH850P/1H-C ASIL D MCU with lockstep cores running at 240 MHz and two Renesas R-Car H3 ASIL B SoCs with four Cortex A57, four Cortex A53, one Cortex R7, one IMP-X5, and one IMG PowerVR GX6650 GPU.

**FIGURE 1:**
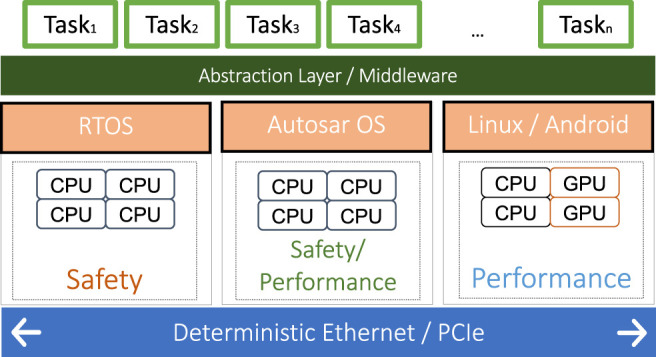
High-level platform model.

Figure 1 presents a high-level view of the ADAS platform, which is similar to the platform described in (Marija Sokcevic, 2020). Each host can run a different operating system depending on the safety and performance requirements. Each such OS can have a different scheduling policy, ranging from fixed-priority (AUTOSAR (Bunzel, 2011)) to table-driven or dynamic priority scheduling (typically in safety RTOSes). However, there is a growing tendency to use a table-driven static schedule execution due to the compositionality and isolation properties (Lukasiewycz et al., 2012; Sagstetter et al., 2014; Mehmed et al., 2017; Ernst et al., 2018), i.e., tasks that are already scheduled are not influenced by new tasks being added to the system. In order to provide a common execution environment and hardware abstraction, a middleware layer, e.g., the MotionWise (TTTech Computertechnik AG, 2018) layer, is running on top of each operating system. The middleware layer also ensures portability of software functions to be located according to their execution and safety requirements (Niedrist, 2018). Moreover, the middleware layer provides the capability to execute tasks according to a table-driven pre-computed schedule independent of the underlying OS dispatching mechanisms, which ensures temporal isolation (Mehmed et al., 2017). Hence, in this paper, we focus on creating static schedules for the table-driven dispatching mechanism of such ADAS systems.

Tasks performing software functions of different criticality levels communicate with each other both on- and off-chip through different means. On-chip communication is usually done through buffers, message passing, or shared memory, while off-chip communication is achieved either through PCIe or TSN. The safety-critical communication also has to adhere to stringent timing requirements and has to be aligned to the execution schedule of the real-time tasks. For example, when PCIe is used, a message sending cost has to be taken into account when scheduling the respective communicating tasks. When using time-aware switched Ethernet technologies like TSN, the schedule of the messages has to be aligned to the execution of the tasks, and the end-to-end latency requirements comprising both task execution and message transmission have to be met.

We model an ADAS platform as a graph A(V,E) . A vertex $v_{i}\in V$ is either an end system (ES) that performs computations, the set of all ESes denoted with ES , or a TSN switch (SW), their set being denoted with SW . The edges E are the communication links. Each ES $v_{i}\in \mathrm{ES}$ has a set of computation cores $C_{i}$ .

### 2.2 TSN

Time-Sensitive Networking (IEEE, 2016b) addresses the need to have more determinism and real-time capabilities over standardized Ethernet networks. To achieve this, TSN defines a series of amendments to the IEEE 802.1Q standard, as well as stand-alone mechanisms and protocols (e.g., 802.1ASrev). TSN has already seen adoption in the industrial domain and is becoming increasingly relevant in the automotive domain. The main mechanisms out of the TSN ecosystem that we consider in this paper are the clock synchronization protocol IEEE 802.1ASrev (IEEE, 2016a), which provides a synchronized clock reference, and the timed-gate functionality of IEEE 802.1Qbv (IEEE, 2015) bringing scheduled communication capabilities on the egress ports of devices. The timed-gate mechanism is essentially a shaping gate that forwards selected message streams from each egress queue according to the transmission schedule encoded in so-called Gate-Control Lists (GCL). A TSN stream is defined by a payload size, a talker (sender), one or more listeners (receivers), and optional timing requirements in terms of jitter and latency. The global schedule synthesis has been studied in (Craciunas et al., 2016; Dürr and Nayak, 2016; Pop et al., 2016; Serna Oliver et al., 2018) focusing on enforcing deterministic transmission, temporal isolation, and compositional system design for critical streams with end-to-end latency requirements.

A communication link is modeled as a directed edge is represented by two vertices $\left[v_{a},v_{b}\right]\in E$ . All physical links in the system are bidirectional and so for each $\left[v_{a},v_{b}\right]\in E$ , there exists a $\left[v_{b},v_{a}\right]\in E$ with the same properties except that source and destination are swapped. In TSN-based systems, a directional virtual edge is created for each core, i.e., the edge [*v*_*a*_, *v*_*a*_] is added to E . Each link $\left[v_{a},v_{b}\right]\in E$ has a set of attributes. We denote with [*v*_*a*_, *v*_*b*_].*s* the transmission bandwidth of the link, [*v*_*a*_, *v*_*b*_].*c* denotes the number of queues in associated egress port.

### 2.3 Application Model

On top of this platform, many different software functions implemented by different vendors must be integrated and deployed. It is crucial that software functions (which may be tested independently) can be integrated with other software functions compositionally. The system is composed of applications (called tasks or runnables) that are either pre-assigned to cores or must be assigned by the scheduling algorithm. Tasks have real-time requirements, both in terms of execution (offset, deadline, jitter) as well as temporal dependencies arising from task chains (defined below). We model the applications as a set of *n* periodic tasks, Γ = {*τ*_*i*_∣1 ≤ *i* ≤ *n*}, similar to the model in (Liu and Layland, 1973). A task *τ*_*i*_ is defined by the tuple (*σ*_*i*_, *r*_*i*_, *ϕ*_*i*_, *C*_*i*_, *T*_*i*_, *D*_*i*_) with *σ*_*i*_ representing the core, *C*_*i*_ denoting the worst-case execution time (WCET), *T*_*i*_ the period, *r*_*i*_ the earliest release time, *ϕ*_*i*_ the initial offset/displacement of task arrival times and *D*_*i*_ the relative deadline of the task under the assumption that *D*_*i*_ ≤ *T*_*i*_. Each real-time task *τ*_*i*_ yields an infinite set of instances (jobs) *τ*_*i*,*k*_, *k* = 1, 2, … (Buttazzo, 2011, p. 80). Tasks can be preempted at any time instant on a timeline with macrotick granularity given by the underlying OS capabilities.

If a task *τ*_*i*_ is pre-assigned to a core, then its core *σ*_*i*_ will be given. Otherwise, we decide their assignment to a specific core, in that the *σ*_*i*_ of a task *τ*_*i*_ can take any value from a finite set of core values $C_{i}$ . The assignment of tasks to cores is captured by the mapping function $M:\Gamma \to C_{i}$ .

The scheduling allows preemption, i.e., a schedule table can be constructed such that a task is interrupted by another task and then resumes its execution. Currently, tasks cannot migrate at run time after they have been assigned to a core, but in the future, we envision that task migration, when done correctly with respect to the deterministic timing behavior, will allow even better resource utilization.

Tasks may exchange messages. A message is modeled as a flow (stream) $\Phi_{i}\in L_{\Phi }$ , which has a period Φ_*i*_.*T* which is the same as its sender and receiver tasks, a deadline Φ_*i*_.*D*, which can be arbitrary but smaller than or equal to the period Φ_*i*_.*T*, a message size Φ_*i*_.*P*, and a fixed route Φ_*i*_.*r*. Messages are transmitted as frames. If a message exceeds the maximum transmission unit (MTU) of 1,500 bytes defined for standard Ethernet, then the message is split into $k=\left\lceil \frac{\Phi_{i}.P}{1500}\right\rceil$ fragments. The message is split such that each resultant frame is its maximum size until the last, which has the last bytes. A frame $\theta_{i,m}^{\left[v_{a},v_{b}\right]}$ is an instance of the frame on the link [*v*_*a*_, *v*_*b*_]. It is associated with the *i*th flow and has sequence number *m*. A frame has a transmission duration of $\theta^{\left[v_{a},v_{b}\right]}.L$ microseconds on the link [*v*_*a*_, *v*_*b*_] where it is transmitted. A frame can have a maximum payload of MTU 1,500 bytes plus the 42 bytes Ethernet header.

### 2.4 Timing Constraints

Each task may have implicit timing constraints arising from the task definition and explicit design parameters related to arrival offsets and/or deadline requirements. Hence, a task must execute periodically with the given period *T*_*i*_, and in each period, it must finish its worst-case execution *C*_*i*_ within the defined deadline *D*_*i*_, starting after the earliest release time *r*_*i*_. In addition, tasks may also have jitter requirements, i.e., constraints on the variance of execution of consecutive period instances (Buttazzo, 2011, p. 81-82), due to control loop considerations (Di Natale and Stankovic, 2000). We denote the jitter requirements of a task *τ*_*i*_ with *J*_*i*_ and the observed jitter *j*_*i*_, i.e., the maximal deviation of both starting and finishing times for any two consecutive task instances are bounded by *J*_*i*_.

Other timing requirements are related to message passing between tasks, where the communication latency has to be considered. The most complex set of timing requirements come from the so-called task (or event) chains (c.f. (Becker et al., 2016a)). A task chain specifies that at least one instance of every task in the given task chain list has to be executed in the specified order within a given maximum end-to-end *reaction latency*. These chains also have a priority, *p*_*i*_, which can be used for optimization criteria. Since the tasks in the chain can be on different hosts/cores, the communication needs have to be included in the end-to-end latency considerations. In a PCIe backbone, the latencies between communicating tasks are modeled and enforced as an additional delay after executing the sending tasks. In the case of TSN, the frame schedule must be aligned to the task execution to ensure that the messages are sent after the sending task has been executed and before the receiving task starts. Please note that tasks in the chains may have different activation patterns and periodicity, i.e., we are considering multi-rate chains (c.f. (Becker et al., 2017)).

**FIGURE 2:**
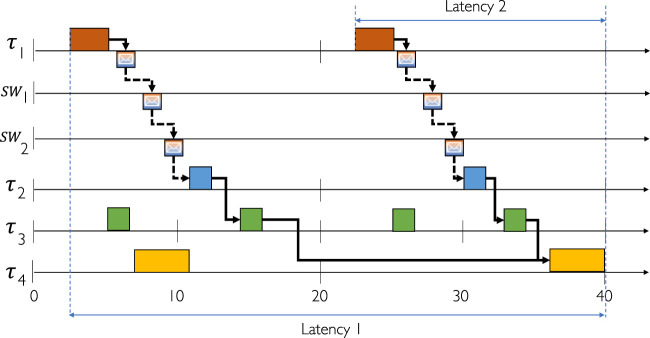
Task chain example with TSN communication.

We give a simple example of a task chain in Figure 2 composed of 4 tasks, a source (*τ*_1_), two processing tasks (*τ*_2_, *τ*_3_) and a sink (*τ*_4_) with periods of 20 *ms*, 20 *ms*, 10 *ms* and 20 *ms*, respectively. The critical communication between *τ*_1_ and *τ*_2_ is done off-chip through the TSN network over two switches (*SW*_1_ and *SW*_2_) since *τ*_1_ and *τ*_2_ are running on different SoCs. For the purposes of this illustration, tasks *τ*_2_, *τ*_3_, and *τ*_4_ are located on the same core, and the communication is assumed to be in 0-time. From each instance of the source, there needs to be a succession of instances of the other tasks in the right order such that the latency is not exceeded. It is allowed that an instance of the processing or sink tasks merges multiple signals. In the example, the sink merges the signal from two execution instances of the processing task *τ*_3_. The communication frames from *τ*_1_ to *τ*_2_ have to be scheduled in such a way that the message is forwarded through the switches and arrives at *τ*_2_ before the respective instance of *τ*_2_ executes. The latency of the communication needs to be also included in the total end-to-end latency.

Let $L_{ℵ}$ denote the set of task chains, where a task chain is given by the tuple *ℵ*_*i*_ = ({*τ*_1_ ≺…≺ *τ*_*k*_}, *L*_*i*_, *p*_*i*_) with *L*_*i*_ being the allowed end-to-end latency and *p*_*i*_ ∈ [0, 1] is the priority. For a chain ${ℵ}_{i}=\left(\left\{\tau_{1}≺\ldots ≺\tau_{k}\right\},L_{i},p_{i}\right)\in L_{ℵ}$ , we formalize the correctness condition for the in-order execution and end-to-end latency requirement as follows:

$$\begin{array}{cc} & \forall \tau_{1,x},x\in \left\{0,\ldots ,\frac{hp_{i}}{T_{1}}\right\}:\exists \left\{y_{2},\ldots ,y_{k}\right\},y_{j}\in \left\{0,\ldots ,\frac{hp_{i}}{T_{j}}\right\},\forall j\in \left\{2,k\right\}\\ & \text{ such that}\\ & start\left(\tau_{2,y_{2}}\right)\geq end\left(\tau_{1,x}\right)\wedge end\left(\tau_{k,y_{k}}\right)-start\left(\tau_{1,x}\right)\leq L_{i}\wedge \\ & \left(\forall j\in \left\{2,k-1\right\}:start\left(\tau_{j+1,y_{j+1}}\right)\geq end\left(\tau_{j,y_{j}}\right)\right), \end{array}$$ (1)

where *hp*_*i*_ is the hyperperiod of the chain *ℵ*_*i*_ calculated as the least common multiple of the periods of the tasks in the respective chain and interfering tasks.^1^ and *start*(*τ*_*i*,*j*_) and *end*(*τ*_*i*,*j*_) denote the start and end of the execution of the job *τ*_*i*,*j*_, respectively.

If there is communication over the TSN network between any two tasks in the chain, the TSN network schedule needs to reflect several correctness conditions. Firstly, the correctness conditions from (Craciunas et al., 2016) for generating GCL schedules need to be fulfilled in order to have correct and deterministic frame transmission over IEEE 802.1Qbv TSN devices. In addition to the technological constraints of standard full-duplex Ethernet networks, a deterministic timing of frames is enforced in (Craciunas et al., 2016) through so-called frame/flow isolation constraints. In the timed-gate mechanism of IEEE 802.1Qbv, the transmission schedule applies to the entire traffic class (as opposed to individual frames like in, e.g., TTEthernet). Therefore, the queue state has to be known and deterministic in order to ensure that the right frames are sent at the right time. Hence, the isolation conditions in (Craciunas et al., 2016) enforce that a correct GCL schedule isolates frames of different flows either in the space domain by placing them in different egress queues or in the time domain, preventing frames of different flows from being in the same queue at the same time. Secondly, implementation or network-specific correctness conditions need to be fulfilled. Here we mention the synchronization error and the microtick of the timeline. The synchronization protocol defined in IEEE 802.1As-rev ensures a common clock reference; however, individual clocks may still have a bounded time differential towards the clock reference. The maximum of all the individual bounded clock errors is called the network precision (*δ*). Furthermore, the (hardware) realization of the required state machines defined in, e.g., IEEE 802.1Qbv implementing the TAS mechanism also has a certain overhead, resulting in a minimum mandatory spacing of scheduled events (called microtick or link granularity). The microtick or link granularity defines the fastest rate at which schedule events can be processed by the TSN hardware and hence, the granularity of the TSN scheduling timeline. Thirdly, the GCL schedule and, more specifically, the schedule offsets of the frame transmission need to be aligned to the task schedule. For example, a message transmitted between two tasks within a chain has to be scheduled for sending after the execution of the sending task and has to arrive before the receiving task executes. Additionally, the communication latency, which adds to the chain’s overall end-to-end latency, has to be within the given latency requirements.

For a given mapping *M*, we denote the schedule table with S . In this table, each task *τ*_*i*_ has a list of offsets *O*_*i*_ on its core *σ*_*i*_. The first offset in *O*_*i*_, denoted with *ϕ*_*i*_, captures the initial offset of *τ*_*i*_’s arrival time within the schedule, and the rest of the offsets in *O*_*i*_ are the times when task *τ*_*i*_ resumes its execution if preempted. The schedule table S also contains the GCLs, which are captured via an offset $\theta^{{\left[v_{a},v_{b}\right]}_{i,m}}.\phi$ , where *i* and *m* are the flow and the frame instance, respectively, and [*v*_*a*_, *v*_*b*_] is the link on which the frame is transmitted. Figure 2 shows a Gantt chart, which is a visual representation of a schedule table.

## 3 Problem Formulation

The scheduling algorithm needs to find an assignment of unassigned tasks to cores such that the tasks are schedulable on each assigned core concerning their timing constraints (offsets, deadlines, and jitter) as well as concerning the task chain requirements. Moreover, since there is communication either between individual tasks or between tasks in a task chain, the scheduling algorithm also needs to find a schedule for the deterministic communication backbone that respects the required maximum latencies.

As an input to our problem we have 1) the ADAS platform A and 2) the applications, denoted by the set of applications Γ, including the task chains $L_{ℵ}$ and all the mapping and timing constraints. We are interested in determining 1) a mapping *M* of tasks to the cores of the platform and 2) a static schedule S of tasks on each core, such that the task deadlines and their jitter, as well as end-to-end constraints on task chains are satisfied. We consider a constant delay for the PCIe communication backbone as part of a task’s WCET. For the TSN backbone, we assume that the flows and their routing are given (e.g., using the shortest path), and the schedule S also contains the offsets of frames, i.e., we also 3) determine the TSN GCLs.

## 4 Mapping and Scheduling Strategy

The presented in the previous section is a combination of the problems in (Pop et al., 2016) and (McLean et al., 2020). Both problems are complex scheduling problems, and the decision problem associated with them have been thoroughly investigated in the literature. (Sinnen, 2007) and (Garey and Johnson, 1979) prove it to be in the NP-complete class by reducing it to the known 3-PARTITION and PARTITION problems. With the assumption that *P* ≠ *NP*, the scheduling problem cannot be solved efficiently by a polynomial-time algorithm. In our initial investigation of the problem, we have implemented a solution using Optimization Modulo Theories (OMT), which is an extension of Satisfiability Modulo Theories (SMT), based on the work in (Craciunas and Serna Oliver, 2016). However, the OMT/SMT approach has not been able to find solutions due to the complexity of the problem. The increasing complexity of ADAS platforms renders such mathematical programming approaches, including Integer Linear Programming (ILP) (Craciunas and Serna Oliver, 2016), infeasible in practice. It is expected that ADAS platforms, which already have the complexity of an entire in-vehicle electronics system (TTTech Computertechnik AG, 2018), will grow to a scale of thousands of functions with hundreds of complex event chain requirements. For such intractable problems, researchers have proposed the use of problem-specific heuristics and metaheuristics (Burke and Kendall, 2005), as an alternative to exact optimization methods which have exponential running times. Several metaheuristic approaches have been presented in the literature (Burke and Kendall, 2005), and the challenge is to identify the right metaheuristic for our problem. Metaheuristics aim to find a good quality solution in a reasonable time but do not guarantee that an (optimal) solution will always be found. Based on the review of the related work, we have decided to implement a combination of heuristics for scheduling, based on List Scheduling (Sinnen, 2007) and a *simulation* of the Earliest Deadline First (EDF) (Craciunas and Serna Oliver, 2016) scheduling algorithm. For the mapping, we have decided to compare the Simulated Annealing (SA) and Genetic Algorithm (GA) metaheuristics, which have been shown in the literature to be a promising approach for task mapping problems.

### 4.1 Solution Overview and Cost Function

**FIGURE 3:**
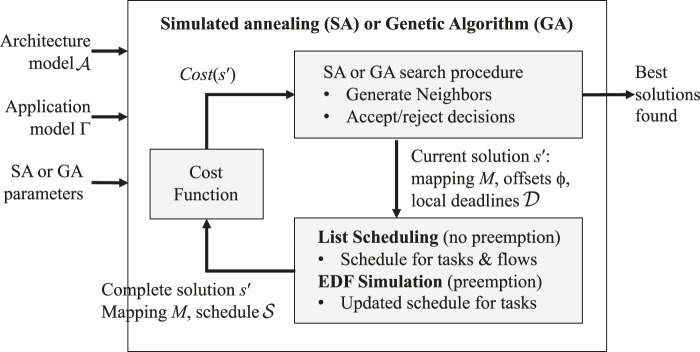
Solution overview.

An overview of our optimization strategy is illustrated in Figure 3. The metaheuristics (SA or GA) decide the mapping *M* via an iterative search which generates neighboring solutions from the current solution, see “SA or GA search procedure” box in the figure, see the details in Section 4.2. The schedule S of flows and messages is decided by a combination of scheduling heuristics, i.e., List Scheduling (LS) and Earliest Deadline First (EDF), see the box with LS and EDF in the figure, and the details in Section 4.3 and Section 4.4, respectively. Finally, the quality of a solution is evaluated using a *cost function*, see “Cost Function” box in the figure and the discussion later in this section. We present our proposed solution for TSN-based systems. The same solution is used for PCIe-based systems, with the observation that the flows are not considered, i.e., they are modeled as an overhead added to the sending task’s WCET.

We use an LS-based heuristic to jointly schedule the flows and the tasks involved in communication, presented in Section 4.3. Once the communicating tasks and flows are scheduled, we use an Earliest Deadline First (EDF)-based scheduling heuristic to add the tasks not involved in communication across cores and optimize the schedule, also by introducing design-time “preemption”, i.e., task splitting, see Section 4.4. We then check if the schedule adheres to the timing requirements imposed by the jitter and task chain constraints. The EDF scheduling heuristic introduces design-time task preemption by *simulating* at design time an EDF scheduling policy parameterized by task offsets and local deadlines. The heuristics receive as an input the mapping of tasks to cores. The LS heuristic is controlled by the tasks and flow offsets (*ϕ*_*i*_ ≥ 0), which are the earliest times a task can be started, or a flow can be sent. The EDF heuristic is controlled by both the offsets and local deadlines D , see the arrow in Figure 3 from the “SA or GA search procedure” box and the LS and EDF box below.

The mapping and the controlling parameters (offsets, deadlines) for the scheduling heuristics are determined by the metaheuristics, as part of their search procedure. We have developed two metaheuristics, one based on Simulated Annealing (SA), see Section 4.2.1 and one based on a Genetic Algorithm (GA), see Section 4.2.2. Both metaheuristics modify the mapping of tasks *M*, the task and flow offsets (*ϕ*_*i*_ ≥ 0), and deadlines D , to find an optimal solution with respect to the cost function. The novelty in our approach is that our metaheuristics make use of the different dimensions that influence task execution, i.e., task mapping, task offsets, and task deadlines, to converge to a near-optimal solution faster than traditional approaches.

The cost function (*Cost*), defined in Eq. 2, captures both a minimization objective with respect to the end-to-end latency of task chains and penalties representing constraint violations given by the application. The function has two cases, 1) a value if the solution configuration meets all the timing constraints and 2) a combination of static and dynamic penalties if one or more timing constraints are violated.

$$Cost\left(s\right)=\left\{\begin{array}{cc} \frac{\underset{{ℵ}_{i}\in L_{ℵ}}{\sum }\frac{l_{i}}{L_{i}}\cdot p_{i}}{\left|L_{ℵ}\right|}\cdot w_{1}\; & if\chi \left(s\right)=true\\ w_{1}+\rho_{ℵ}+\rho_{D}+\rho_{J}\; & if\chi \left(s\right)=false \end{array}\right.$$ (2)

During the search, the metaheuristics do not reject the invalid solutions. Instead, we “penalize” an invalid solution by increasing its cost function value to be larger than the values for valid solutions in the hope of driving the search towards valid solutions. A solution is *invalid* if one of the three constraints is violated: 1) There is a task or flow which does not meet its deadline, i.e., the worst-case response time *f*_*i*_ of a task *τ*_*i*_ is larger than its deadline *D*_*i*_ (or the worst-case delay of a flow is larger than its deadline). 2) There is a chain *ℵ*_*i*_ which has an end-to-end latency *l*_*i*_ that is greater than its allowed latency *L*_*i*_. 3) There is a task *τ*_*i*_ which has a jitter *j*_*i*_ greater than maximum allowed jitter *J*_*i*_, see the notations in Section 2.4. We capture each of these constraint violations using a separate penalty term, which is zero if the constraint is not violated: 1) *ρ*_*D*_ for deadline violations, 2) *ρ*_*ℵ*_ for chain latency violations and 3) *ρ*_*J*_ for jitter violations. Hence, if the sum of these penalty terms is zero then the solution is valid, i.e., all constraints are satisfied and thus all penalty terms are zero. We capture this situation with a test defined by *χ*(*s*) = *ρ*_*ℵ*_ + *ρ*_*D*_ + *ρ*_*J*_ > 0. *χ*(*s*) is true if the solution is valid, i.e., case (1) in Eq. 2, and false if the solution is invalid, corresponding to case (2).

Let us first discuss case (1) when the solution is valid. In this case, the value of the cost function which has to be minimized is defined as the average weighted distance of the measured end-to-end latency *l*_*i*_ over the imposed constraint *L*_*i*_, of all task chains. Basically, the smaller the chain latencies, the smaller the term. When we sum up $\frac{l_{i}}{L_{i}}$ we also multiply with the chain’s priority *p*_*i*_ to capture the relative importance of chains. The resulted summation is divided with the number of chains $\left|L_{ℵ}\right|$ . Note that when a solution is valid, *l*_*i*_ ≤ *L*_*i*_. The divisions of *l*_*i*_ by *L*_*i*_ and of the summation by $\left|L_{ℵ}\right|$ are intended to normalize the cost function term. In addition, we multiply the thus resulted term with a static penalty weight *w*_1_. We will discuss the use of weights shortly when we cover case (2).

**FIGURE 4:**
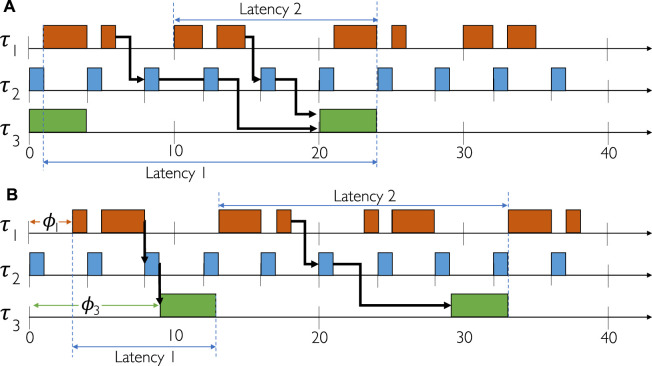
Schedule optimization. **(A)** End-to-end task chain latencies not satisfied. **(B)** End-to-end task chains latencies satisfied.

Let us consider the example in Figure 4 where we have three tasks and one chain on a single core. The details are given next to the figure, with the note that the allowed end-to-end latency of the chain *L*_1_ is 20, and its priority *p*_*i*_ is 1.0. Figure 4A shows a valid solution, whereas Figure 4B an invalid one. For Figure 4A we have *l*_1_ = 20, hence the cost function is $\frac{20}{20}\cdot 1.0\cdot w1=1\cdot w1$ . Let us now discuss the cost function for the case 2) when a solution is invalid. In that case, *ρ*_*ℵ*_ + *ρ*_*D*_ + *ρ*_*J*_ will be greater than zero. To this term, we add *w*_1_ to ensure that any invalid solution will be rated worse relative to that of any valid solution. That is, it adjusts the score such that the minimal penalty value is higher than that of any feasible solution, thereby preventing the search process from accepting any invalid candidate over a valid one. As discussed, we use *ρ*_*D*_ to penalize deadline violations, *ρ*_*ℵ*_ to penalize chain violations and *ρ*_*J*_ to penalize jitter violations.

The values of these penalty functions are dynamic, i.e., a larger value is used bigger constraint violations. We first define the *ρ*_*ℵ*_ from Eq. 3 in detail and then discuss the other two penalties, which are similarly constructed. The relative importance of these penalties are determined by the weights *w*_2_, *w*_3_ and *w*_3_. *ρ*_*ℵ*_ in Eq. 3, measures the weighted average of end-to-end violation. The violation of a chain *ℵ*_*i*_ is defined as the difference between its highest observed chain latency *l*_*i*_ and its end-to-end constraint *L*_*i*_.

$$\rho_{ℵ}=\frac{\underset{{ℵ}_{i}\in L_{ℵ}}{\sum }\frac{min\left(L_{i},max\left(0,l_{i}-L_{i}\right)\right)}{L_{i}}}{\left|L_{ℵ}\right|}\cdot w_{2}$$ (3)

If *l*_*i*_ is smaller or equal to *L*_*i*_ then the chain constraint is satisfied and the term is zero. We discuss here the case when the constraint is not satisfied, i.e., *l*_*i*_ > *L*_*i*_. When *l*_*i*_ > *L*_*i*_ the *max* operator will return *l*_*i*_ − *L*_*i*_. To normalize the penalty value, we clamp any observed violation *l*_*i*_ − *L*_*i*_ to the interval [0, *L*_*i*_] using the *min* operator and divide by *L*_*i*_, hence the term in the summation will be in the interval [0, 1]. We divide the sum with the number of chains $\left|L_{ℵ}\right|$ and then multiply with the static weight *w*_2_. Let us illustrate this with the example in Figure 4B, where the latency *L*_*i*_ = 20 for chain *ℵ*_1_ is violated, i.e., *l*_*i*_ = 23. Hence, $\rho_{ℵ}=\frac{\frac{min\left(20,max\left(0,23-20\right)\right)}{20}}{1}\cdot w_{2}=\frac{3}{20}\cdot w_{2}$ .

Likewise, the additional deadline and jitter costs (*ρ*_*D*_ and *ρ*_*J*_) is listed by Eqs. 4, 5, respectively. Here *ρ*_*D*_ measures the weighted average of deadline violations with a violation range clamped in the interval [0, *D*_*i*_]. The deadline violation of a task or flow *i* is denoted as the difference between the maximal relative finishing time of all of *i*’s instances *f*_*i*_ and the relative deadline *D*_*i*_.

$$\rho_{D}=\frac{\underset{i\in \Gamma }{\sum }\frac{min\left(D_{i},max\left(0,f_{i}-D_{i}\right)\right)}{D_{i}}}{\left|\Gamma \right|}\cdot w_{3}.$$ (4)

Finally, *ρ*_*J*_ measures the weighted average of jitter violations. We define the jitter violation of a task *τ*_*i*_ as the difference between the maximal observed jitter *j*_*i*_ and the threshold *J*_*i*_. The violation range is then clamped in the interval [0, *J*_*i*_].

$$\rho_{J}=\frac{\underset{\tau_{i}\in \Gamma }{\sum }\frac{min\left(J_{i},max\left(0,j_{i}-J_{i}\right)\right)}{J_{i}}}{\left|\Gamma \right|}\cdot w_{4},$$ (5)

In Eqs. 2–5, we list *w*_1_, *w*_2_, *w*_3_ and *w*_4_ as static weights designed to capture the importance of the respective violation with the following constraints: *w*_2_ ≥ *w*_1_, *w*_3_ ≥ *w*_1_, *w*_4_ ≥ *w*_1_. The constants were determined based on manual experimentation and observations, with *w*_1_ through *w*_4_ set to 10,000, 40,000, 10,000, and 60,000, respectively. Please note that there are no optimal values for the weights, since they have to be adapted to the application domain, criticality definitions and design goals of the respective use-case.

### 4.2 Metaheuristics

The SA and GA metaheuristics aim to iteratively optimize solutions by randomly changing existing solutions *s* to create new solutions *s*′ and evaluate them by using the cost function. They take as input the platform model A and the applications Γ and return the best solution s* found according to the cost function. Both start from an initial solution *s*_0_. Metaheuristics can start from any initial solution, even a random one. However, in our case, we have developed a *Greedy* algorithm to generate the initial solution. With SA and GA the mapping is optimized during the search. With Greedy, the mapping is decided constructively as follows. For each task, considering the processor affinity constraints (that restrict the mapping to specific processors), we iterate through all cores and identify the core with the lowest utilization. The utilization of a task is its WCET divided by its period. The utilization of core is the sum of all task utilizations on that core. We then map the task to the respective core. This ensures a balanced utilization of cores in the initial solution. Once the mapping is decided, we use the same LS and EDF scheduling heuristics to schedule the tasks and flows, see Section 4.3 and Section 4.4, respectively. However, with Greedy, the input parameters to the scheduling heuristics, such as offsets for both LS and EDF and local deadlines for EDF, are not optimized. Thus, we consider that the offsets are all set to zero and the local deadlines are set to the absolute deadlines.

#### 4.2.1 Simulated Annealing

We first describe a Simulated Annealing (SA)-based metaheuristic approach, which uses an EDF-based heuristic to solve the task scheduling problem. Simulated Annealing is a heuristic method that aims to optimize solutions by randomly selecting a candidate solution in the neighborhood of the current one (Burke and Kendall, 2005). The SA algorithm accepts a new neighbor solution if it is better than the current one. Moreover, a worse solution can be accepted with a certain probability given by the cost function *Cost* and the *cooling scheme* defined by an initial temperature, *t*_*s*_ and a cooling rate *cr*, specifying the rate at which the temperature drops with each iteration.

A new candidate solution *s*′ (also called neighbor) is generated starting from the current solution *s* by performing design transformations (also called *moves*) on *s*. We use three moves, described in the following. *AdjustDeadline* adjusts the deadline of a single randomly selected task. Only tasks that failed at complying with the jitter constraints are potential candidates for this move. Note that the deadlines in D are used to control the resulting EDF schedule. We do not change the relative deadline *D*_*i*_ of the task, which is one of its timing constraints. For a task *τ*_*i*_, AdjustDeadline will modify the deadline used by EDF to schedule *τ*_*i*_, such that it is lower or equal to *D*_*i*_. We check for each resulted schedule that all timing constraints are satisfied. *SwapTasks* swaps the core mapping of two randomly selected tasks, considering the imposed mapping constraints. For example, if the task has a processor affinity, the swapping is done within the cores of the particular processor. Only tasks that are allowed to swap are considered, meaning only tasks without a predefined core assignment. Offset and Deadline adjustments are reset to zero for both tasks when performing this action. Finally, the utilization/core load is not considered, and as such, this action might overload one of the cores. *AdjustOffset* changes the offset of a randomly selected task or flow.

The function that generates neighbor solutions is implemented as a simple state machine, allowing moves mentioned earlier to be chosen randomly. Various probability assignments for these moves were tried, and, based on observations from performed experiments, a uniform distribution has been chosen for all actions.

#### 4.2.2 Genetic Algorithm

GA is a multi-objective optimization heuristic inspired by evolutionary biology (Deb et al., 2000). We 1) encode each solution (*chromosome*) as an array where each entry (*gene*) contains information on the mapping, offset and deadline of a task/flow and 2) randomly initialize *N* *individuals*. We then 3) evolve some selected candidates by using 4) recombination and 5) mutation. Finally, 6) the evolved candidates with better *fitness* will replace the parent population. As mentioned, GA is a multi-objective metaheuristic. This means that the *fitness* is captured with several cost function values, i.e., *ρ*_*ℵ*_ for chains, *ρ*_*D*_ for deadlines and *ρ*_*J*_ for jitter constraints, see the discussion in Section 4.1. This is in contrast to SA, which collapses all these terms into a single cost function value, as defined in Eq. (2). Steps 3) to 6) are repeated until the allotted time is exhausted.

Several crossover operators have been proposed in the literature, and we have implemented: uniform order-based crossover (OX), (OX) using 2-point and using 1-point approaches (Syswerda, 1991), Partially-Mapped Crossover (Goldberg and Lingle, 1985), Cycle-Based Crossover (Goldberg and Lingle, 1987) and Alternating-Position Crossover (Larranaga et al., 1997). Based on our experiments, we have decided to employ a standard uniform crossover. Regarding mutation, for each gene in the chromosome, we compare a randomly generated number with a “probability of mutation”, and if this number is smaller, then this position is mutated. The probability of mutation has been determined using ParamILS (Hutter et al., 2009) as discussed in Section 5. To select parents, we sort the “population” using the “non-dominated” sorting method from (Deb et al., 2000). Half the population is kept as parents, and to create new individuals, two random parents are picked until all individuals have been created.

### 4.3 Joint Flow and Task Scheduling

**FIGURE 5:**
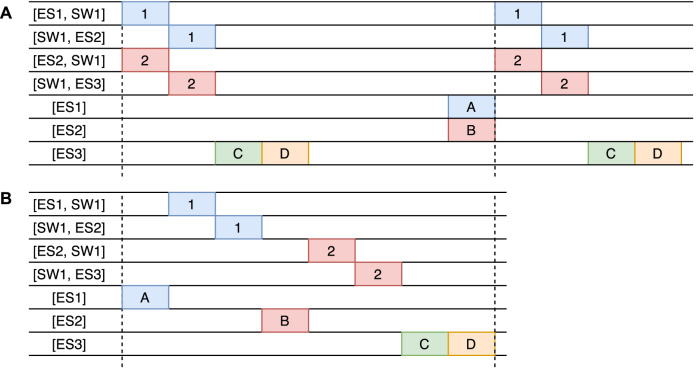
Scheduling approaches, **(A)** is a schedule where flows 1 and 2 are scheduled before taking tasks into account, **(B)** is the optimized scenario where task and flows are scheduled concurrently.

One approach to the task and flow scheduling problem is to solve the problems separately and then fit them together. This is a reasonable approach when the two sub-problems do not form a circular dependency. In our case, however, the two scheduling problems are closely linked together. Let us consider the example in Figure 5, where we have a topology of three end systems (*ES*_1_ to *ES*_3_) and one switch (*SW*_1_). We have four tasks *τ*_*A*_, *τ*_*B*_, *τ*_*C*_, and *τ*_*D*_ with precedence constraints [*τ*_*A*_ ≺ *τ*_*B*_ ≺ *τ*_*C*_ ≺ *τ*_*D*_] that form a chain. *τ*_*A*_ needs to be executed on *ES*_1_, *τ*_*B*_ on *ES*_2_ and *τ*_*C*_ and *τ*_*D*_ on *ES*_3_. Task *τ*_*A*_ sends a message to *τ*_*B*_, and *τ*_*B*_ sends a message to *τ*_*C*_. For illustration purposes, the task WCETs and the transmission times of message frames on links are a single time unit, and network precision and macrotick are ignored. The period of all tasks is 8 time units, and the chain latency is also 8 time units.

If the messages are scheduled first, then the solution of the flow would look as shown in Figure 5A. This schedule minimizes the flow latencies, but since task *τ*_*B*_ must receive message 1 and send message 2, the schedule contains a lot of idle time; hence, the chain latency becomes 13 time units. Note that messages 1 and 2 scheduled at the beginning of the schedule are sent by tasks *τ*_*A*_ and *τ*_*B*_ from the previous period. The same issue exists if the tasks are scheduled without any knowledge of the network. However, if both task scheduling and message scheduling are optimized concurrently, then an optimized solution, shown in Figure 5B, can be produced. This reduces the chain latency substantially to 8 time units from 13 in Figure 5A, meeting thus the task chain latency constraint.

In this section, we propose a joint flow and task scheduling heuristic based on List Scheduling (LS) (Sinnen, 2007). LS is a widely used task scheduling heuristic that is known to obtain good quality solutions when determining static schedules for tasks on multiprocessors. We have re-purposed LS for jointly scheduling flows and tasks. Our LS is inspired by the individual flow scheduling heuristic from (Raagaard and Pop, 2017), which uses variants of ASAP (As Soon As Possible) and ALAP (As Late As Possible) scheduling. Both of these are a special case of the List Scheduling heuristic (Sinnen, 2007). Our LS is more general, scheduling both flows and tasks. This LS can use offset and ordering parameters to control the placement of frames, which is not considered in (Raagaard and Pop, 2017).

LS receives as input the architecture A , applications Γ and the solution *s* generated by the outer metaheuristic, containing the mapping *M*, offsets *ϕ* and deadlines D . LS returns a partial schedule table $S^{\prime }$ covering the hyperperiod of Γ and including all flows and those communicating tasks involved in sending and receiving the flows. The joint scheduling is achieved by adding those tasks involved in communication as “virtual flows” and the cores where they are mapped for execution as “virtual links”. Then, precedence constraints are added to ensure that a frame cannot be sent before its sending task has finished executing, and a receiving task cannot start before its input frames have arrived. Hence, in the following, flows and links also denote tasks and cores, respectively. Note that not all tasks are involved in the communication over TSN. Tasks that are not sending/receiving flows are added to the schedule in a subsequent step using the EDF-based heuristic from Section 4.4.

Algorithm 1 ScheduleFlow (*θ*, *ϕ*). Schedules a flow *θ* as soon as possible (ASAP), considering its offset *ϕ* given by the metaheuristic. All frames in the flow have initialized lower and upper bounds to − *∞*, *∞*, respectively.
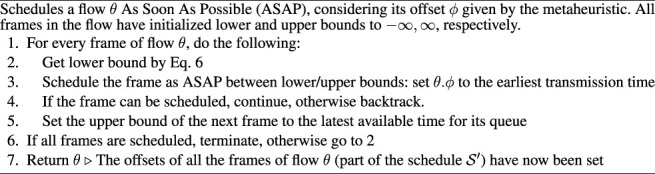

Similar to (Raagaard and Pop, 2017), LS starts with an empty timeline and iteratively schedules one flow at a time. The metaheuristic specifies the order in which flows are chosen. Similar to (Raagaard and Pop, 2017), the flows are chosen according to their deadlines since flows with tight deadlines are the hardest to schedule and therefore should be picked first. The tie-breaker for the ordering is given by the flow period. Each flow is scheduled using the ScheduleFlow procedure in Algorithm 1 such that the end-to-end latency is minimized. The termination condition for the LS is that either a schedule has been found for all flows or the current iteration does not produce a feasible schedule with respect to the flow deadline.

We now examine the steps of Algorithm 1 in more detail. In step 1 frames are retrieved in the order given by Eq. 7, see Section 4.3.3 for an explanation of how the next frame is determined. In step 3, the frame offset *ϕ* is set to the earliest time in a *feasible region*, greater than the *lower bound*, determined in step 2. Section 4.3.2 and Section 4.3.1 present how we define and determine the feasible regions and the lower bound, respectively. If the algorithm reaches a state where a frame cannot be scheduled, e.g., there is not enough space, then it needs to find another solution. This search is done by backtracking: In step 4 backtracking is done by increasing the lower bound to the latest available time which is less than the frame offset found in step 3, then rescheduling the previous frame, see Section 4.3.4.

#### 4.3.1 Lower Bound

The lower bound for the LS algorithm, inspired by (Raagaard and Pop, 2017), is calculated using Eq. 6. $\theta_{i,m}^{\left[v_{x},v_{a}\right]}.\left(\phi +L\right)$ is used as shorthand for $\theta_{i,m}^{\left[v_{x},v_{a}\right]}.\phi +\theta_{i,m}^{\left[v_{x},v_{a}\right]}.L$ , and where *ϕ* is the frame offset and *L* is the frame transmission duration. This equation ensures that the assigned offset fulfills the link congestion and flow transmission constraints, i.e., that frames must be fully received before being transmitted. The *link congestion* constraint does not allow two or more frames to be sent on the same link at the same time. The *flow transmission* constraint restricts the sending of a frame to be after the reception and buffering of that frame in the switch. The link congestion and flow transmission constraints result in a minimum possible end-to-end latency for flows. This lower bound is influenced by the route of the flow as well as the flow characteristics. The following equation, introduced in (Raagaard and Pop, 2017), captures the lower bound on $\theta_{i,m}^{\left[v_{a},v_{b}\right]}$ , when considering the previous frame on the same link, $\theta_{i,m-1}^{\left[v_{a},v_{b}\right]}$ , and the same frame on the previous link, $\theta_{i,m}^{\left[v_{x},v_{a}\right]}$ .

$$\underline{\phi }\left(\theta_{i,m}^{\left[v_{a},v_{b}\right]}\right)=\left\{\begin{array}{cc} \theta_{i,m}^{\left[v_{a},v_{b}\right]}.\phi \; & ifm=1\wedge \left[v_{a},v_{b}\right]=\Phi_{i,s}\\ \theta_{i,m-1}^{\left[v_{a},v_{b}\right]}.\left(\phi +L\right)\; & ifm>1\wedge \left[v_{a},v_{b}\right]=\Phi_{i,s}\\ \theta_{i,m}^{\left[v_{x},v_{a}\right]}.\left(\phi +L\right)+\delta \; & ifm=1\wedge \left[v_{a},v_{b}\right]\neq \Phi_{i,s}\\ \max\left(\theta_{i,m-1}^{\left[v_{a},v_{b}\right]}.\left(\phi +L\right),\right.\;\\ \;\;\left.\theta_{i,m}^{\left[v_{x},v_{a}\right]}.\left(\phi +L\right)+\delta \right)\; & Otherwise. \end{array}\right.$$ (6)

#### 4.3.2 Feasible Regions

When a flow is scheduled, each frame will *block* those queues and the network links where it is scheduled. The *feasible region* for a frame, similar to (Raagaard and Pop, 2017), is a set of intervals where the frame can be scheduled without violating the feasibility of the existing partial schedule. The algorithm relies on feasible regions to find out where the frames can be scheduled without interfering with other frames. Since frames can have different periods, this complicates the search for space where the frame can be scheduled.

We introduce two operations that the feasible region implement, i.e., queue *blocking*, and *searching* for the feasible region of a frame. *Blocking* is used when a frame is scheduled in a known feasible region, and *searching* is used when the algorithm is searching for an appropriate place for a frame. *Blocking* happens at most once for every flow scheduled, while *searching* can happen several times, depending on how hard it is to schedule a frame. In order to minimize the time in search, the following method of constructing the feasible regions is used.

If a frame $\theta_{i,m}^{\left[v_{a},v_{b}\right]}$ is scheduled on the network, then it will block the egress queue $\Phi_{i}^{\left[v_{a},v_{b}\right]}.\rho$ from $\theta_{i,m}^{\left[v_{a},v_{b}\right]}.\phi$ to $\theta_{i,m}^{\left[v_{a},v_{b}\right]}.\phi +\theta_{i,m}^{\left[v_{a},v_{b}\right]}.L$ . When we need to determine if a frame of another period interferes with the other frame, we have to check that it does not interfere in the whole hyperperiod. Instead of determining this each time we have to check for a new frame, we create a feasible region for each different period in the network. The feasible regions of other periods are then blocked using the BlockQueues procedure from Algorithm 2.

Algorithm 2 BlockQueues $\left(\theta_{i,m}^{\left[v_{a},v_{b}\right]}\right)$ . Procedure for blocking time slots inside the feasible regions for a frame instance *θ*_*i*,*m*_ on a link [*v*_*a*_, *v*_*b*_]
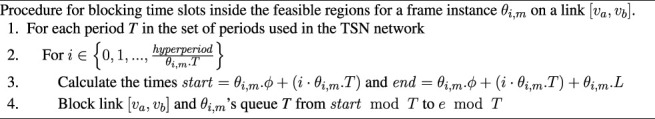

**FIGURE 6:**
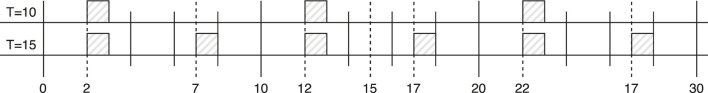
Blocking times of a frame with a period of 15 *ms* considering another frame with a period of 10 *ms* over their hyperperiod.

Step 4 of Alg. 2 does the queue blocking. It takes a *start*, *end*, and a frame, and blocks the frame’s queue and link in that interval, where mod is the modulo operator. If *start* > *end*, then the queue is blocked in the intervals [0, *end*[ and [*start*, *Q*_*T*_], where *Q*_*T*_ is the period of the queue. An example of the blocking is illustrated in Figure 6, where we show on a link [*v*_*a*_, *v*_*b*_] how the feasible region of a frame instance *θ*_*i*,*m*_ is blocked. Let us assume that an earlier frame in the same queue and link had a period of 10 *ms* and our frame’s period is *θ*_*i*,*m*_.*T* = 15 *ms*. Both frames have an offset of 2 *ms* and their transmission times are 1 *ms*. Frame instance *θ*_*i*,*m*_ cannot use the time slots where the earlier frame has been scheduled, at every 10 *ms*, the first row in Figure 6. In addition, we also need to block those times where, if *θ*_*i*,*m*_ is scheduled periodically with a period of 15, runs the risk of conflicting with the other frame with a period of 10. For example, *θ*_*i*,*m*_ cannot be scheduled at time 7, because its next occurrence at 7 + 15 = 22 would conflict with the other frame that periodically is scheduled every 10 *ms* with an offset of 2, i.e., 2 + 10 + 10 = 22. The second row in Figure 6 shows the times blocked by Algorithm 2 for our example with two frames.

#### 4.3.3 Getting the Next Frame

The LS heuristic schedules the frames in the order specified by Eq. 7:

$$NextFrame\left(\theta_{i,m}^{\left[v_{a},v_{b}\right]}\right)=\left\{\begin{array}{cc} \theta_{i,1}^{\text{LCL}}\; & if\;\;bLNL\wedge bLS\\ \theta_{i,m+1}^{\text{FNL}}\; & if\;\;bLNL\\ \theta_{i,m}^{\text{NL}}\; & otherwise, \end{array}\right.$$ (7)

where *LCL* is the last “virtual link” (core), *FNL* is the first network link, *NL* is the next link, *bLNL* is true when frame is on the last network link, and *bLS* is true if the frame is the last frame in the flow. The NextFrame function is valid for all frames except the last “virtual frame”, where *NextLink* is not defined.

**FIGURE 7:**
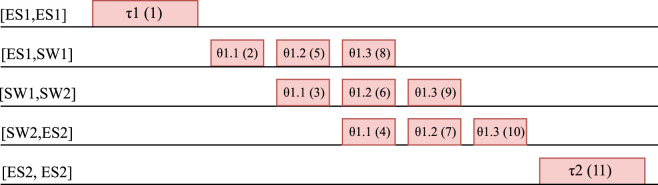
How tasks and flow frames are scheduled together. The order is indicated by the number in parenthesis.

LS starts from the first “virtual frame” (sender task), and goes through each frame and ends with the last “virtual frame” (receiving task). The idea is to allow backtracking only to change the last scheduled frame. We illustrate an ordering in Figure 7, where the order is indicated in parenthesis inside the rectangles representing tasks and frames. In Figure 7 we have a setup where a task *τ*_1_ on *ES*_1_ modeled as a “virtual frame” on the “virtual link” [*ES*_1_, *ES*_1_] sends a flow *Φ*_1_ of size 3xMTU, which hence has to be split in three frames *θ*_1_.1, *θ*_1_.2 and *θ*_1_.3, to a task *τ*_2_ on *ES*_2_.

Figure 7 shows the order in which Eq. 7 will visit the frames. Note that by using this order and converting tasks to “virtual frames” on “virtual links” we can treat the tasks and frames together and schedule them jointly. Thus, the frames *τ*_1_ and *τ*_2_ on “virtual links” [*ES*_1_, *ES*_1_] and [*ES*_2_, *ES*_2_], respectively, are “virtual frames” (tasks), hence they are scheduled as tasks without concern for MTU-size limits. However, the flow *Φ*_1_ has to be split into frames *θ*_1_.1 to *θ*_1_.3, which are then scheduled as frames on the physical links. The idle times in the schedule in Figure 7 between each frame are due to the link granularity and synchronization, which have been considered for this example.

#### 4.3.4 Backtracking

**FIGURE 8:**
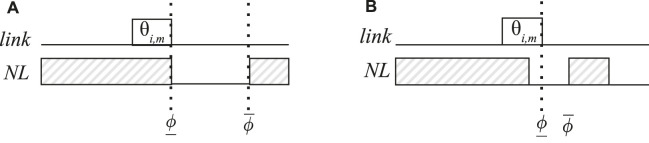
Visualization of lower bound and upper bounds. The hatched areas are already filled by other frames, such that the non-hatched areas form the feasible region. **(A)** Case where the next frame can be scheduled in the feasible region. **(B)** Case where there is not enough space is available to schedule the frame and backtracking will be used to move the frame forward.

When the LS heuristic schedules a frame instance *θ*_*i*,*m*_, it sets the upper bound of the frame instance $\theta_{i}^{\mathrm{NL}}.\bar{\phi }$ on the next link *NL* to the latest time available in the queue of the next link *NL*. When we schedule the frame instance $\theta_{i,m}^{\mathrm{NL}}$ there can be two situations, visualized in Figures 8A,B. In Figure 8 we show the previous *link* where the frame instance *θ*_*i*,*m*_ was transmitted, and the next link *NL* where the frame instance $\theta_{i,m}^{\mathrm{NL}}$ has to be transmitted next. On the link *NL* we show with hatched rectangles the times where $\theta_{i,m}^{\mathrm{NL}}$ cannot be transmitted, e.g., because other frames are being transmitted.

Case (1) is when we have enough space to send $\theta_{i,m}^{\mathrm{NL}}$ on *NL*, depicted in Figure 8A. The frame can then be placed into the queue as early as the constraints allow. Conversely, case (2), is when there is not enough space for the frame on *NL*, as shown in Figure 8B. In this case, we use *backtracking*. We first need to check later times on *NL* when there is space to transmit $\theta_{i,m}^{\mathrm{NL}}$ . This is achieved by increasing the lower bound of *θ*_*i*,*m*_ on the previous link such that $\theta_{i,m}^{\mathrm{NL}}$ will have to be delayed, i.e., scheduled at a later time. If we are unsuccessful in pushing $\theta_{i,m}^{\mathrm{NL}}$ later, we need to push also *θ*_*i*,*m*_ later, which is achieved by going back on the links where *θ*_*i*,*m*_ was transmitted, and delaying the frames. This is the backtracking process, which continues going back on the previous links and delaying the frames until we are able to find space for all of them. If such a space cannot be found, it means that the frame cannot be scheduled, hence this solution is infeasible. The metaheuristics will hopefully then guide the search in their outer loop to other solutions where the frame can be scheduled.

### 4.4 EDF Simulation for Schedule Synthesis

The List Scheduling heuristic from Section 4.3 has scheduled all the flows and the corresponding communicating tasks, resulting in a partial schedule $S^{\prime }$ . We propose the EDF-based heuristic in Algorithm 3 for scheduling the rest of the non-communicating tasks and optimizing the schedule. *EDFScheduleSynthesis* receives as input the architecture A , applications Γ the partial solution *s* containing the mapping *M*, offsets *ϕ*, deadlines D and partial schedule $S^{\prime }$ . The schedule synthesis heuristic from Algorithm 3 is based on *simulating* Earliest Deadline First (EDF) scheduling, similar to (Craciunas et al., 2014; Barzegaran et al., 2020). EDF is a scheduling algorithm (Buttazzo, 2011) that prioritizes tasks at each time instant depending on their deadlines, i.e., the one with the earliest deadline will get control of the CPU. Given the task WCETs, offsets, and deadlines, the partial schedule $S^{\prime }$ , the complete schedule table S is generated by simulating how EDF would execute tasks until the hyperperiod. For a given mapping, offsets, and deadlines, EDF will always produce the same schedule. We optimize the schedule produced by the EDF simulation by allowing the metaheuristics in an outer loop to change the mapping *M*, offsets *ϕ*_*i*_ of each task *τ*_*i*_ and deadlines D , and by allowing preemption for the tasks. The EDF simulation is implemented using the *discrete-event simulation* (DES) paradigm, where the operation of a system is seen as a discrete sequence of events in time, and an *event* captures the change of state in the system at a particular moment in time.

Algorithm 3 EDFScheduleSynthesis (A,Γ,s) . Schedules the tasks that are not involved in the communication on top of the partial schedule $S^{\prime }$ produced by Algorithm 1, creating the final schedule S 
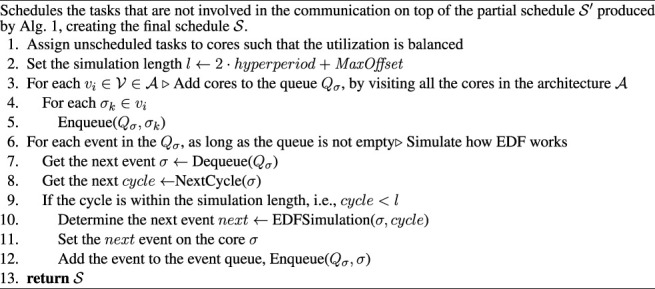

We start by assigning all tasks to their respective cores (step 1 in Algorithm 3). All tasks without a task mapping will be mapped according to a best-fit strategy with respect to utilization, i.e., balancing the processor and core utilization. We run the simulation for a length *l* (set in step 2), after which the schedule will repeat itself. *l* is defined by 2 ⋅*hyperperiod* + *MaxOffset*, where hyperperiod is determined as the Least Common Multiple of all tasks in Γ and the *MaxOffset* is the maximum over all offsets *ϕ*_*i*_ (Leung and Merrill, 1980). The iteration over the simulation length *l* is done in the steps 6–12. The current time is captured by *cycle*, and we advance the time to the *next* event that needs to be simulated.

The EDF simulation is performed per core *σ* (step 10), and we use a queue *Q*_*σ*_ containing all cores from all processors, ordered by the earliest event that needs to be simulated. To start the simulation, steps 3–5 add cores to the queue *Q*_*σ*_, by visiting all the cores in the architecture A .

The next event to be simulated is determined by taking the head of the queue *Q*_*σ*_ (Dequeue) and calling NextCycle. NextCycle is called for a core *σ* and returns the time *cycle* when the next event of interest for scheduling occurs on that core. Our NextCycle implementation skips unnecessary cycles, i.e. when no events of interest to the scheduling occur. It does so by progressing towards the nearest event defined by either releasing a task from the waiting queue, choosing the task with the earliest deadline first from the ready queue, completing a task, or allowing preemption to occur on a certain break point defined by a parameter called *macrotick* for each core. The macrotick defines the preemption granularity. The macrotick is set such that it allows preemption, under the constraint that the overhead due to context switches on each processor should be low, see (Aichouch et al., 2013; Craciunas et al., 2014; Zuepke et al., 2015) for a discussion.

We add cores to be simulated in *Q*_*σ*_ only if we are still within the simulation length *l*. The algorithm stops when there are no cores to be simulated (*Q*_*σ*_ is empty). The EDF simulation logic is taking place in the EDFSimulation function, called at line 10, which simulates up to the *next* event, which is returned. The product of EDFScheduleSythesis is then a recording of all occurred events, from which we can derive the schedule table S of the current solution *s*. As mentioned, LS schedules jointly the flows and those tasks involved in the communication. EDF schedules the tasks not scheduled so far, i.e., those which are not involved in inter-core communication. The EDF algorithm allows task preemption, which in our case means that tasks in the schedule can be split at design-time. This optimizes the schedule table, as the scheduling search space becomes larger and latencies and jitters can be further optimized compared to the case such task splitting is not allowed. In addition, EDF will be allowed to split the communicating tasks already added to the partial schedule $S^{\prime }$ by the LS heuristic if such task splitting will not result in constraint violations.

We illustrate the EDF approach via the example in Figure 4, consisting of an architecture with a single processor with two cores, *σ*_1_ and *σ*_2_, both of which having a macro tick of 1 *ms*. The depicted application is modelled by the task set Γ = {*τ*_1_, *τ*_2_, *τ*_3_}. In this example all tasks have a jitter constraint of 0, meaning that they have to start in each period instance at the same time in relation to the start of the period. The tasks are defined as *τ*_1_ = (*σ*_0_, 0, *ϕ*_1_, 4, 10, 10), *τ*_2_ = (*σ*_0_, 0, *ϕ*_2_, 1, 4, 4) and *τ*_3_ = (*σ*_1_, 0, *ϕ*_3_, 4, 20, 20). Furthermore, the set of task chains is defined by $L_{ℵ}=\left\{{ℵ}_{1}\right\}$ , with *ℵ*_1_ = ({*τ*_1_ ≺ *τ*_2_ ≺ *τ*_3_}, 20, 1.0). Please note, that given Eq. 1, only two chain instances are necessary to validate as the hyperperiod of *ℵ*_1_ is 20 *ms*, and the period of *τ*_1_ is 10 *ms*.

Figure 4A depicts a solution, where the jitter and the task chain end-to-end constraints are violated, whereas Figure 4B shows a valid solution. As seen from Figure 4A, *τ*_1_ violates its jitter constraints, as the start (and end) of execution within its periods varies. This is detected when the events for the respective tasks instances are raised. For example, the event triggering the start of execution with respect to *τ*_1,1_ and *τ*_1,2_ differs by 1 *ms*. While the initial offset *ϕ*_*i*_ for all tasks is 0, resulting in *τ*_2,1_ and *τ*_3,1_ starting their execution first, neither are source tasks with respect to *ℵ*_1_. Moving forward, *τ*_1,1_ is started at cycle 1, causing the event to trigger the registration of a task chain instance *ℵ*_1,1_. At cycle 4, *τ*_1,1_ is preempted by *τ*_2,2_ while *τ*_3,1_ completes is execution. Although *τ*_3,1_ is a sink task, and a chain instance has been registered, the instance has yet to receive the completion of *τ*_1,1_ and *τ*_2,*k*_ before it is accepted. That is, the presence of an event from *τ*_2,*k*_ that happens after *τ*_1,1_ must be registered. Subsequently, *τ*_1,1_ completes at cycle 5, allowing the *ℵ*_1,1_ to advance its state, waiting for *τ*_2,3_. Lastly, *τ*_3,2_ finalizes its execution at cycle 23, thus completing *ℵ*_1,1_ with a resulting latency of 23, which incidentally violates the given constraints. The chain instance *ℵ*_1,2_ is registered at cycle 10, and finalizes at cycle 23, yielding a latency of 14. Given that both latency and jitter constraints have been violated, the product of the EDFScheduleSynthesis is not feasible.

However, in an optimized solution, solving the associated violations can be achieved by manipulating the initial offsets *ϕ*_*i*_ for the tasks, as depicted in Figure 4B. Here, the schedule has been altered such that all executions of *τ*_1_ and *τ*_3_ have been deferred by *ϕ*_1_ and *ϕ*_3_. For *τ*_1_, the displacement *ϕ*_1_ solves the jitter constraint violation, because all jobs *τ*_1,*i*_ now start (and end) at the same cycle relative to its period. Finally, *τ*_3_ has been displaced by 9 cycles, such that its initial execution allows *τ*_3,1_ to catch the events from *τ*_2,3_ (and by extension *τ*_1,1_), thus reducing the latency of *ℵ*_1,1_. Likewise, by introducing *ϕ*_1_ for *τ*_1_ the latency of *ℵ*_1,1_ was reduced even further. The combined effect of *ϕ*_1_ and *ϕ*_3_ is full compliance of all constraints with the resulting latencies of 10 and 20 *ms* for *ℵ*_1,1_ and *ℵ*_1,2_, respectively.

## 5 Experimental Results

We have evaluated the proposed solutions in for both network setups, PCIe in Section 5.1 and TSN in Section 5.2. We have used both realistic test cases and synthetic test cases. The synthetic test cases were generated using a tool developed for this purpose (McLean et al., 2019), which derives the desired task properties from the realistic test cases. The test case generation tool was extended to add TSN flows based on the work from (Craciunas and Serna Oliver, 2016). All experiments were conducted on a High Performance Computing (HPC) cluster, with each node configured with 2xIntel Xeon Processor 2660v3 (10 cores, 2.60 GHz) and 128 GB memory. Both SA and GA run on one node at a time.

The choice of parameters for the metaheuristics has been done using ParamILS (Hutter et al., 2009), which performs a stochastic search in the parameter space. ParamILS works by giving it a list of possible values for each parameter to be tuned. The list of values was initially chosen on a broad scale, and then if runs seemed to converge, the range was narrowed. Because the different test case sizes have varying difficulty, parameter tuning was done separately for the different test case sizes. For each type of test case, 10 parallel runs were launched with differing seeds so that more solutions could be discovered (Hutter et al., 2012).

### 5.1 Experimental Results for PCIe-Based Systems

As a first experiment, we were interested in determining our proposed SA’s ability to find near-optimal solutions. We have implemented an exhaustive search that finds the optimal solution; however, we could only do that for small task sets of less than 10 tasks considering an architecture with two cores. Our SA was able to find the same optimal solution in less than 10 s. In the following sets of experiments, determining the efficacy of SA was achieved through a combination of synthetic and realistic test scenarios, benchmarked against two other heuristics: Greedy, presented as the initial solution for the metaheuristics in Section 4.2 and Genetic Algorithm (GA).

#### 5.1.1 Synthetic Test Cases

We were then interested in determining if using an SA meta-heuristic combined with EDF-simulation is a viable solution for finding feasible schedules when confronted with very large task sets. Thus, we have used five test cases, ranging from 100 to 500% in scale, i.e., for *ADAS1x100%* the application contains 151 tasks and 31 chains using a model of the architecture discussed in section 2, whereas with *ADAS1x200%* the architecture would double the number of processors, tasks and task chains. The results are presented in Table 1, with each row representing the results of a task case. A *test case* is a scenario consisting of 30 synthetically generated task sets, with each undergoing 30 trials (runs of SA and GA on the same test case). Thus a single test case, e.g., *ADAS1x100%*, would conduct 900 trials for each algorithm. As the experiment progresses through each case, the algorithms were given additional time due to an inherent increased complexity of the problem (see the *Time* column).

**TABLE 1:** Evaluation results on synthetic test cases.

Test case	Time	Greedy							SA							GA						
		Chains			Jitter			Sched.	Chains			Jitter			Sched.	Chains			Jitter			Sched.
		*Min*	*Avg*	*Max*	*Min*	*Avg*	*Max*		*Min*	*Avg*	*Max*	*Min*	*Avg*	*Max*		*Min*	*Avg*	*Max*	*Min*	*Avg*	*Max*	
ADAS1x100%	1 h	0.97	0.98	1.00	0.58	0.61	0.68	1.00	1.00	1.00	1.00	1.00	1.00	1.00	1.00	1.00	1.00	1.00	1.00	1.00	1.00	1.00
ADAS1x200%	2 h	0.97	0.99	1.00	0.55	0.67	0.75	1.00	0.98	1.00	1.00	0.94	1.00	1.00	1.00	0.98	1.00	1.00	0.71	0.95	1.00	1.00
ADAS1x300%	3 h	0.97	0.99	1.00	0.52	0.64	0.72	1.00	0.97	0.99	1.00	0.70	0.87	1.00	1.00	0.97	0.99	1.00	0.70	0.88	1.00	1.00
ADAS1x400%	4 h	0.97	0.97	0.98	0.52	0.64	0.73	1.00	0.97	0.99	1.00	0.69	0.80	0.88	1.00	0.94	0.99	1.00	0.70	0.81	0.92	1.00
ADAS1x500%	5 h	0.97	0.98	0.98	0.51	0.62	0.70	1.00	0.95	0.98	0.99	0.63	0.78	0.86	1.00	0.95	0.98	1.00	0.64	0.79	0.87	1.00

For each algorithm (Greedy, SA, and GA), we show in the table, under the *Sched.* columns, the percentage of cases (out of the 30 trials) for which the algorithms determine schedulable solutions (all *deadline* constraints are satisfied; 1 means 100%). The columns labeled *Chains* have the percentage of chains out of the total chains, for which the respective algorithm was able to satisfy the end-to-end constraints. Similarly, *Jitter* denotes the percentage of jitter constraints satisfied. These values are presented in terms of minimum, average and maximum considering the 30 runs. Note that the Greedy algorithm is not stochastic and always outputs the same result.

As we can see from Table 1, the Greedy approach has comparatively the worst performance in terms of complying with the constraints. We also see that SA can find schedulable solutions (in terms of deadlines, chains, and jitter constraints) within the allotted time, even when the problem size increases. We see that SA has a drop in finding feasible schedules (from 100% in column *Chains.* for ADAS1x100%, to 63% for ADAS1x500%, and cannot meet the jitter constraints for some of the two largest test cases). We estimate that this is caused by a combination of increased difficulty of the task sets and their constraints, as well the crude method for estimating the time allotted. We observed that both SA and GA obtain similar quality results, with SA being slightly better for smaller test cases and GA doing slightly better for larger test cases. Both metaheuristics (SA and GA) are clearly superior to the mapping heuristic, such as Greedy, when presented with very large task sets.

#### 5.1.2 Realistic Test Cases

For the following evaluation, we were interested in the ability of SA to handle realistic test cases. Thus, we have used three test cases, ADAS1 to ADAS3, which are variants of an anonymized realistic task set currently in use in a series-production vehicle. All test cases have 151 tasks and 31 task chains, but with varying jitter, earliest activation, and macrotick constraints. The experiment was set up such that 30 trials were conducted with SA for each test case; the time limit used is in minutes. As we can see from Table 2, SA can find feasible solutions for all test cases. As the test cases get progressively more difficult from ADAS1 to ADAS3, in terms of timing constraints that need to be satisfied, SA retains its ability to find solutions within the allotted time, albeit at a slightly lower rate. By comparison, we see that the percentage of resolved constraints for the Greedy algorithm decreases similarly and fails on all accounts to find feasible schedules that meet all the constraints. We have also implemented an approach from the related work (Verucchi et al., 2020), called *DAG*, which constructs a Directed Acyclic Graph (DAG) from the input task set. The constructed DAG can handle the multiple periods of tasks in the task set and encodes the chain constraints. Such a DAG is built on the fly by our approach when constructing a solution. The DAG approach does not consider “preemption”, i.e., the tasks will not be split when scheduled, and uses List Scheduling instead of EDF for scheduling the DAG. As we can see, the DAG approach from related work is similar to our Greedy approach and significantly under-performs compared to our SA metaheuristic. In addition, it results in fewer jitter constraints satisfied, compared to our Greedy.

**TABLE 2:** Evaluation results on realistic test cases.

Test case	Time	DAG			Greedy			SA						
		Chains	Jitter	Sched.	Chains	Jitter	Sched.	Chains			Jitter			Sched.
						Min	Avg	Max	Min	Avg	Max
ADAS1	3.20	0.90	0.31	1.00	0.81	0.37	1.00	0.97	0.99	1.00	0.95	0.99	1.00	1.00
ADAS2	6.40	0.55	0.10	1.00	0.65	0.21	1.00	0.94	0.99	1.00	0.84	0.99	1.00	1.00
ADAS3	13.20	0.74	0.10	1.00	0.48	0.21	1.00	0.84	0.99	1.00	0.74	0.97	1.00	1.00

### 5.2 Experimental Results for TSN-Based Systems

**FIGURE 9:**
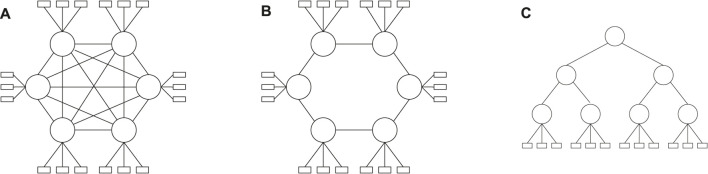
Topologies used for experiments. In each topology, a switch has 3 end systems attached (for tree: Leaf nodes only). **(A)** Mesh topology. **(B)** Ring topology. **(C)** Tree topology with depth = 2.

In this section, we considered that the communication is done via a TSN backbone. We have used synthetic test cases, for which we generated various TSN networks. We have used three different types of graphs with varying degrees of connectivity. The topologies (mesh, ring, and tree) are shown in Figure 9.

The number of end systems, switches, and chains are given in Table 3, similar to the setup used in (Craciunas and Serna Oliver, 2016), except for the number of chains. For each end system, 16 tasks are created, 8 which communicate and 8 which do not. Each communicating task sends a message to another communicating task. Thus, there will be |*ES*|⋅ 4 flows in the network. The utilization is set to be 50% for each end system, 25% of which corresponds to communicating tasks and 75% to the rest. Task WCETs are chosen such that they are divisible with the macrotick and fit within the assigned utilization. Message lengths are chosen at random between 84 and 1,542 Bytes. The macrotick of the end systems is set to 250 *μ*s, and the granularity of the links are set to 1 *μ*s. The speed of links from end systems to switches is set to 100 Mbps and to 1 Gbps between switches.

**TABLE 3:** Number of switches, end systems and chains for each topology and size of test case.

Size	Topology	Switches	End systems	Tasks	Chains	Flows
Small	Mesh, Ring	2	4	64	16	16
	Tree, depth = 1	4	6	96	16	24
Medium	Mesh, Ring	4	16	256	32	64
	Tree, depth = 2	13	36	576	32	144
Large	Mesh, Ring	8	48	768	64	192
	Tree, depth = 3	15	48	768	64	192
Huge	Mesh, Ring	16	192	3,072	128	768
	Tree, depth = 2	43	432	6,912	128	1,728

We have used three sets of randomly chosen periods, all in milliseconds, *P*1 = {10, 20, 25, 50, 100}, *P*2 = {10, 30, 100}, and *P*3 = {50, 75}. We use the shortest paths for routes. The chains are generated with a maximum task length of 15, and a minimum of 2, and lengths are chosen from a uniform random distribution. Two consecutive tasks in a chain must either be on the same end system or be communicating via the TSN network. In order to compare the performance between the two metaheuristics, 10 runs were performed on each of the test cases for a total of 360 runs for each of the metaheuristics. The time given for each size is as follows. 300 s for small test cases, 1,200 s for medium, 4,800 s for large, and 19,200 s for the largest test cases, called “huge”.

**FIGURE 10:**
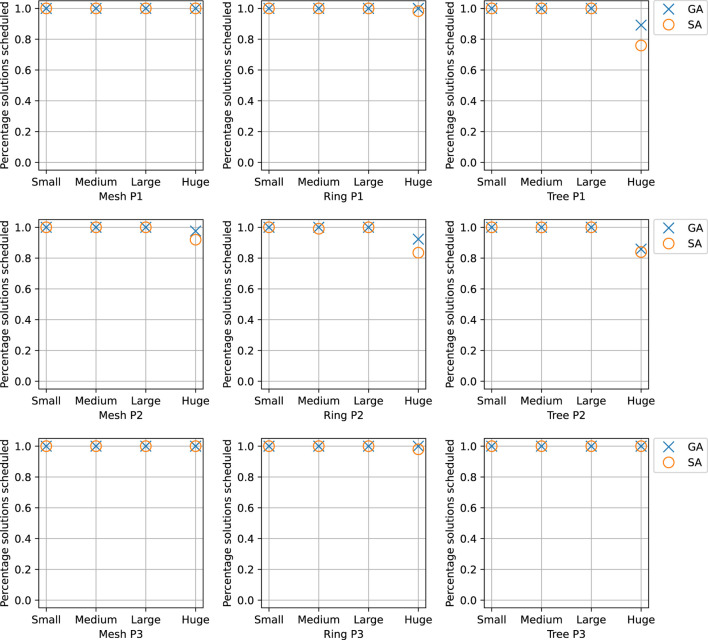
Comparison of SA and GA in terms of percentage of solved solutions, i.e., all the tasks and flows are successfully scheduled and the constraints, e.g., chain latencies, are satisfied.

Figure 10 shows on the *y*-axis the percentage of test cases solved for each size and topology. The results are grouped per topology, mesh, ring, and tree, and for each topology, we use different sets of periods, *P*1 to *P*3. A test case is “solved” if all the requirements are satisfied. On the *y*-axis, 1 means that 100% of the requirements were satisfied, whereas 0 means that no requirements could be satisfied. On the *x*-axis, we show the type of test case, small, medium, large, and huge. As we can see, our GA and SA solutions can successfully solve all the test cases, except for some of the “huge” test cases, especially in the tree topologies, where a few requirements could not be satisfied. In those situations, GA performs better than SA. When considering the cost of the solutions (the value of Eq. 2), we noticed that SA is better than GA in terms of the cost function for small, medium, and large test cases. However, in the huge test cases, GA not only is able to find feasible solutions more consistently but is also able to find solutions of lower cost.

**FIGURE 11:**
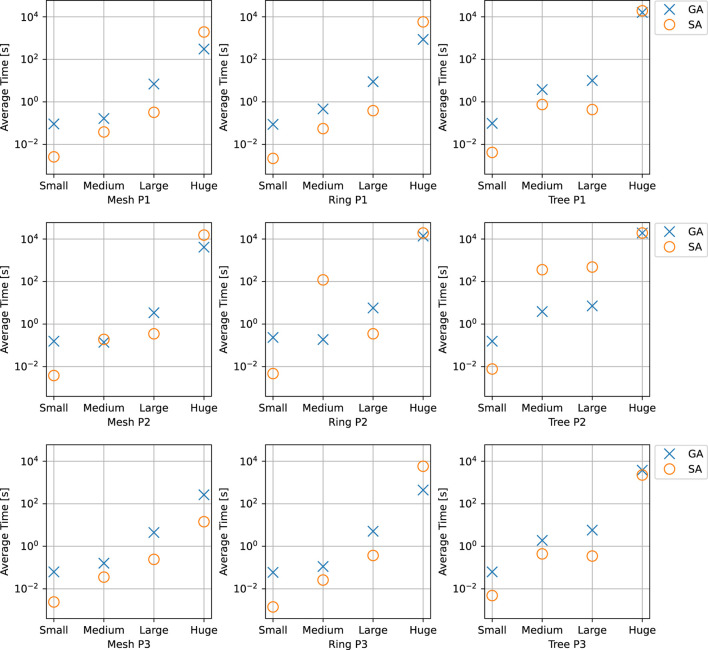
Comparison of SA and GA runtimes when searching for the first feasible solution.

Finally, we were also interested in our approaches’ ability to find a feasible solution as fast as possible. That is, we wanted to determine what is the earliest time when all the requirements are satisfied. Once such a solution is found, the metaheuristics continue the optimization until the time limit is reached. Hence, we modified SA and GA to return once a feasible solution was found. The runtime results are shown in Figure 11. The figure shows that when SA can find a feasible solution for a test case, it generally finds it faster than GA. However, there are situations where GA outperforms SA.

## 6 Conclusion and Future Work

In this paper, we have considered safety-critical ADAS applications mapped on modern multi-processor platforms. The applications are modeled as a set of communicating software tasks with complex timing requirements, e.g., jitter, deadlines, and end-to-end latency bounds on task chains. We have proposed an optimization strategy that, given the application and platform models, determines a mapping of tasks to the cores of the platform and a static schedule of tasks on each core, such that the timing constraints are satisfied. We have also considered a realistic communication backbone implemented using the IEEE 802.1 Time-Sensitive Networking standard, and our optimization derives the schedule tables for the TSN messages.

Our optimization strategy uses metaheuristics (Simulated Annealing and Genetic Algorithm) to explore the solution space, combined with a scheduling heuristic to jointly solve the task and message scheduling problem. The experimental evaluation on several realistic and synthetic test cases has demonstrated that our proposed strategy is able to find solutions that meet the timing constraints at a higher rate than traditional approaches and scales with the growing trend of ADAS platforms.

Our evaluation has shown that SA is superior in finding feasible solutions fast and with a lower cost function value compared to GA, whereas GA outperforms SA for very large TSN-based test cases where. As future work, we want to implement a hybrid multi-objective metaheuristic (Blum and Roli, 2008) that combines SA and GA and considers several optimization objectives, such as reducing the number of task preemptions in order to reduce context switch overhead and reducing the number of switch queues used by the TSN messages.

## Data Availability

The raw data supporting the conclusions of this article will be made available by the authors, without undue reservation.

## Footnotes

1 I.e., tasks that execute on the same core as the tasks in the chain
